# Supplementary figures and images for: Modular pipeline for reconstruction and localization of implanted intracranial ECoG and sEEG electrodes
Source: PLoS One. 2023 Jul 7;18(7):e0287921. doi: 10.1371/journal.pone.0287921 (PMC10328232; doi:10.1371/journal.pone.0287921)

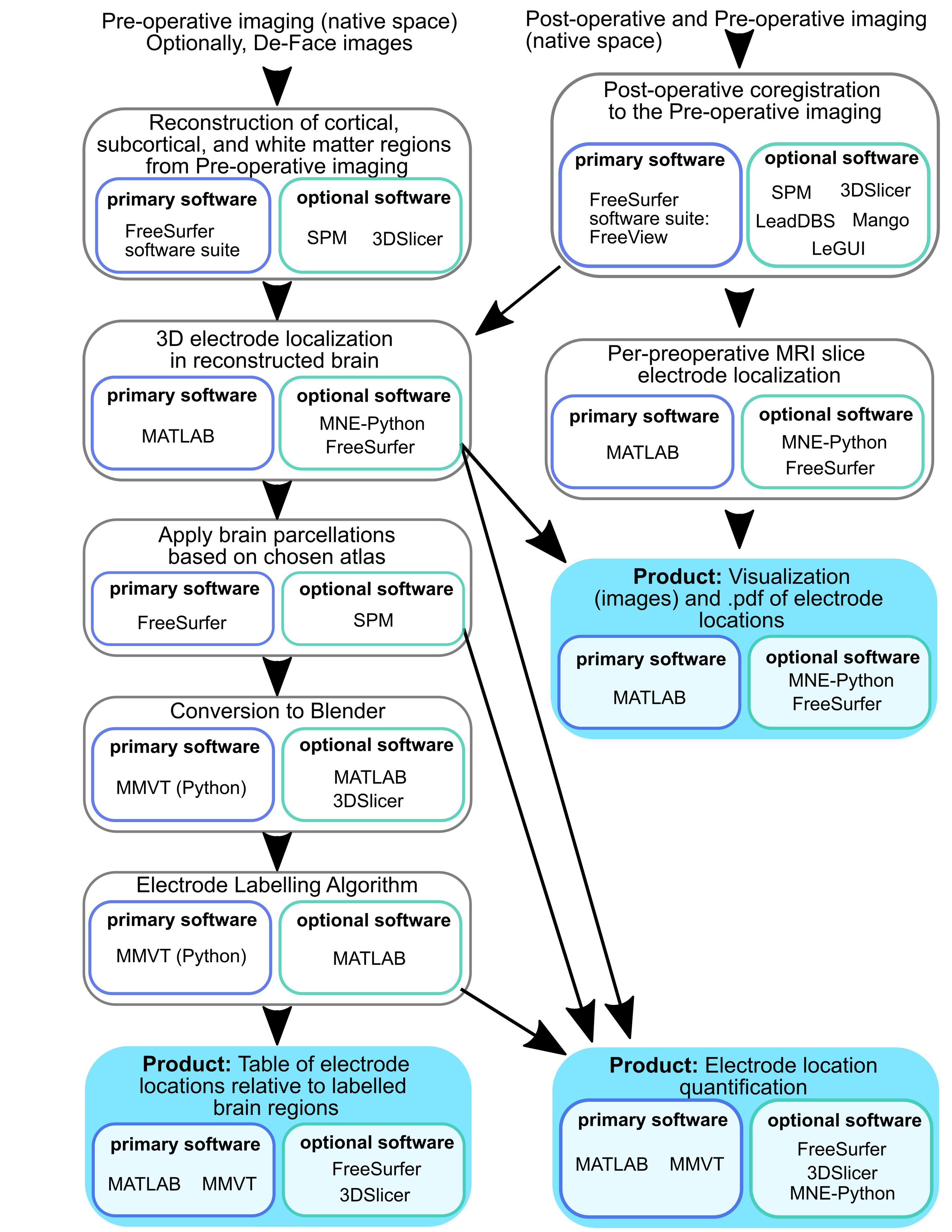

Supplement: S1 Fig — (TIF) [file pone.0287921.s001.tif]

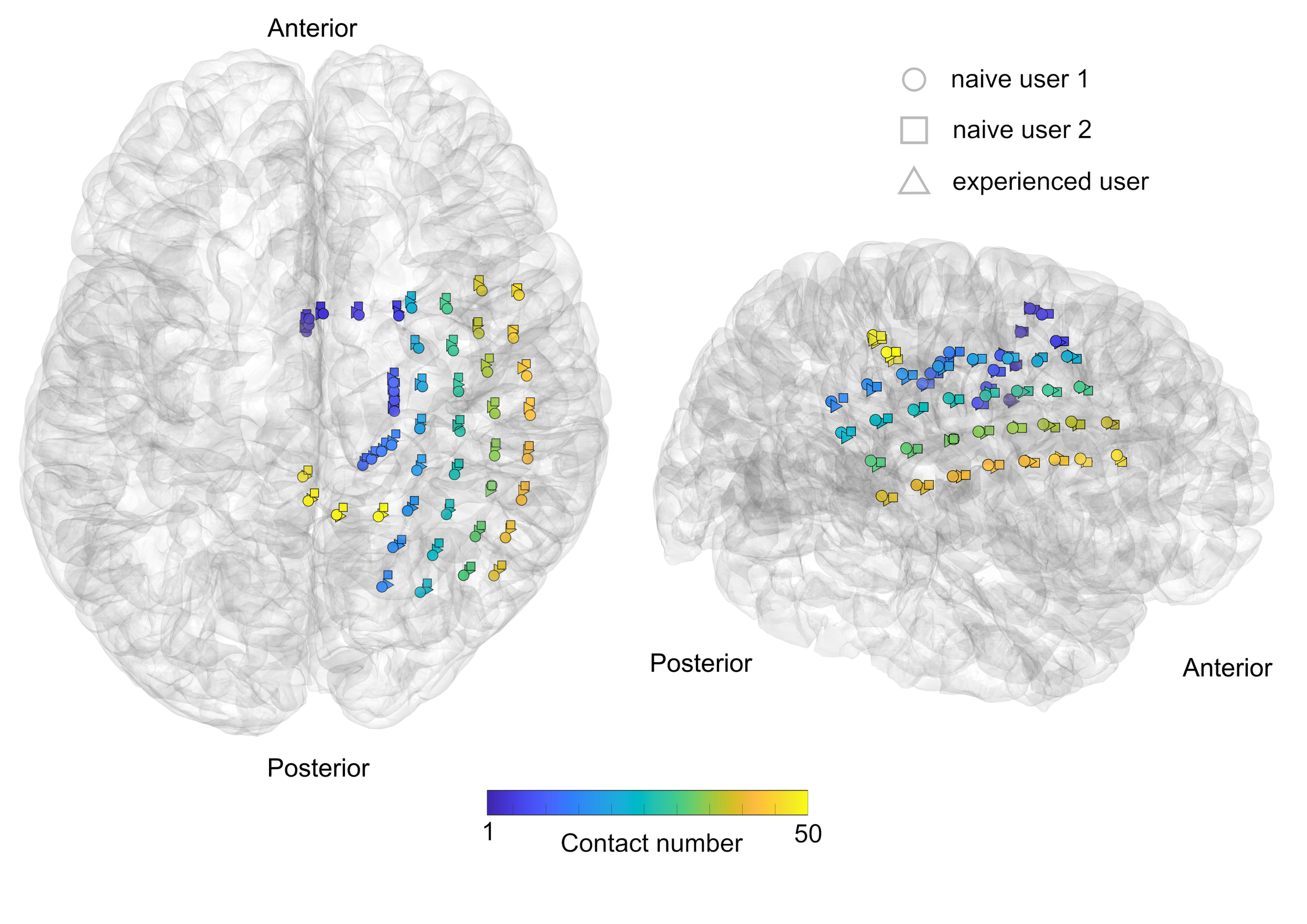

Supplement: S2 Fig — (TIF) [file pone.0287921.s002.tif]
