# Supplementary material for: Modular pipeline for reconstruction and localization of implanted intracranial ECoG and sEEG electrodes
Source: PLoS One. 2023 Jul 7;18(7):e0287921. doi: 10.1371/journal.pone.0287921 (PMC10328232; doi:10.1371/journal.pone.0287921)
Supplement: S1 File — (PDF) [file pone.0287921.s003.pdf]

Modular Reconstruction and Co-registration of Imaging from Implanted ECoG and SEEG Electrodes V.2

Daniel J Soper[1,2], Alex Ross[3], Dustine Reich[1,4], Sydney S. Cash[1,2], Ishita Basu[3], Noam Peled[5], Angelique C. Paulk[1,2]

[1]Center for Neurotechnology and Neurorecovery, Department of Neurology, Massachusetts General Hospital, Boston, MA, USA;
[2]Department of Neurology, Harvard Medical School, Boston, MA, USA;
[3]University of Cincinnati;
[4]Department of Neurology, Brigham and Women's Hospital, Harvard Medical School, Boston, MA 02115, USA.;
[5]Department of Radiology, Massachusetts General Hospital, Boston, MA, USA

Daniel J Soper
Center for Neurotechnology and Neurorecovery, Department of ...

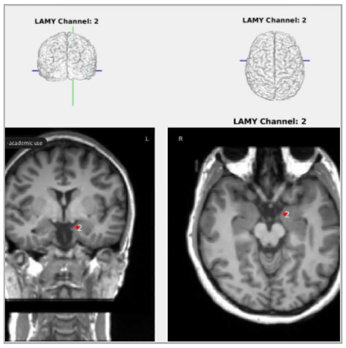

VERSION 2

MAR 23, 2023

OPEN ACCESS

DOI:
dx.doi.org/10.17504/protocols.io.5qpvornedv4o/v2

Protocol Citation: Daniel J Soper, Alex Ross, Dustine Reich, Sydney S. Cash, Ishita Basu, Noam Peled, Angelique C. Paulk 2023. Modular Reconstruction and Co-registration of Imaging from Implanted ECoG and SEEG Electrodes. **protocols.io** https://dx.doi.org/10.17504/protocols.io.5qpvornedv4o/v2Version created by Daniel J Soper

License: This is an open access protocol distributed under the terms of the Creative Commons Attribution License, which permits unrestricted use, distribution, and reproduction in any medium, provided the original author and source are credited

Protocol status: Working
We use this protocol and it's working

Created: Feb 23, 2023

Last Modified: Mar 23, 2023

ABSTRACT

This pipeline involves taking pre- and post-operative images to localized and visualize electrode locations in the case of implanted intracranial electrodes, whether the electrodes penetrate the brain parenchyma (depth, stereoelectroencephalography, sEEG electrodes) or are on the surface of the cortex (strip or grid, electrocorticography, ECoG, electrodes). The pipeline uses a number of different packages in a modular approach that allows use of a subset or all parts of the pipeline depending on need.

MATERIALS

In order to use our pipeline, download MATLAB, FreeSurfer, and iELVis from these links. A Unix operating system (Linux OS or Mac OS) or a Windows computer with a Linux virtual box must be used (particularly with FreeSurfer and MMVT).

Required software to produce the images of electrode locations overlaid on MRIs:
MATLAB
FreeSurfer
iELVis
Fieldtrip

For ELA and for parcellating the brain with less command line coding:
MMVT-lite

Shared MATLAB code that uses these packages are located in the below:

PROTOCOL integer ID:
77536

Keywords: SEEG, ECoG, Reconstruction, Coregistration, Co-registration, Freesurfer, electrodes, epilepsy, co-localization, colocalization, iEEG, DBS

Pipeline Code: Place this in your MATLAB folder

For de-identification:
Fieldtrip

For standardizing the data in iEEG BIDS format:
BIDS starter kit WIKI
BIDS starter kit GitHub

Optional software which would allow you to follow the same pipeline include the below:
Mango: Can be used in place of FreeView
LeadDBS: Reconstructed brains and localizes electrodes for DBS Thalamic Lead implants
LeGUI: Also allows co-registration of pre- and post-operative images
Fieldtrip: Has similar pipeline for coregistration and localization
DCM2NIIX: Can be used in place of *mri_convert*
MMVT: Full version not necessary for this pipeline

Our tutorials for familiarizing yourself with the Terminal, setting up your environment, installing FreeSurfer, and installing MMVT, and much more are found here:
APPENDIX.docx

For examples of our basic pipeline output, please see the two attached examples:
sub-0t3i_Images_small.pdf sub-5o1r_Images_small.pdf

BEFORE START INSTRUCTIONS

This pipeline requires a Linux or Mac operating system (OS) to run FreeSurfer and MMVT. Our instructions here will be specific to Linux, but the same principles apply to a Mac OS. In general, the programs we use (MATLAB and FreeSurfer especially) have comprehensive installation and tutorials, but we will include bare-bones guides to get you started. Please look at Appendix I if you are unfamiliar with using the terminal. See the Appendix II-VII on instructions for checking that your system is correctly configured and installing FreeSurfer. Examples of the pipeline's output are in the Materials section.

Step 1 requires DICOM retrieval tool
Steps 2-4 require FreeSurfer
Step 5-6 require iELVis, MATLAB, Fieldtrip, and our pipeline code on Github
Step 7 requires MMVT-lite

Retrieving Images and Saving Files

1

Retrieve Images

Note

May require the installation of a DICOM viewer like Mango (see Materials)

When retrieving imaging as DICOMs, you can download the entire imaging series or just the sequence of interest, whichever is easiest for your setup. If there is not a T1 MRI with high enough resolution, a T2/FLAIR image with good resolution can supplement (but not replace!) the bad T1 in the FreeSurfer reconstruction process (see Step 2.2).

1.1 Download pre-op MRI imaging. MRI requirement:

- T1 (MPRAGE, SPGR, Ax T1 NAV, ect.) with as many slices as possible, good resolution between grey and white matter, and ideally without contrast (Step 1.2, Panel A).

1.2 Download post-op imaging.

- Ideally post-operative CT (Panel C), although post-operative MRI (Panel B) works as well.

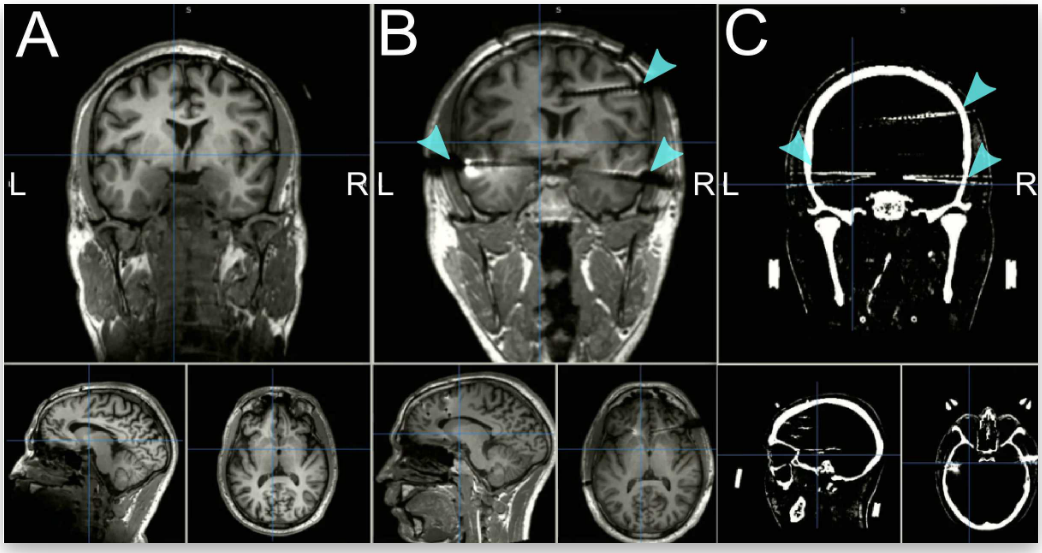

1.3 Convert imaging to compressed NifTI (.nii.gz).

- (Recommended) Download Mango, open image or images folder, "Files > Save As" into a compressed NifTI (nii.gz).
- Alternatively, use FreeSurfer's *mri_convert* function to create NifTI files for both MRI and CT sequences of interest.
- If you run into any issues, *dcm2niix* may be a good backup program for converting

sequences (see Materials).

FreeSurfer Output

2 **FreeSurfer Output**

Once your imaging scans are selected, move the pre-op NifTI file into the folder required by FreeSurfer installation (usually somewhere like ~/home/freesurfer/subjects). During this step, you will open a terminal (right click and select "Open Terminal") and run the commands which make all the 3D files, cortical parcellations, and subcortical segmentations of the input brain. **From here on, any instance of "id##" is the name and number of the subject you are currently running through the pipeline. You must replace every "id##" with your subject name**.

Note

Skip this step if you require only the 2D visuals output. Skipping this step is not possible for grid and strips because a 3D model of the brain will be required to adjust the electrode locations in Snap_to_Grid.m.

2.1 Make a ../freesurfer/subjects/**id##** folder and move the pre-op NifTI file into this folder

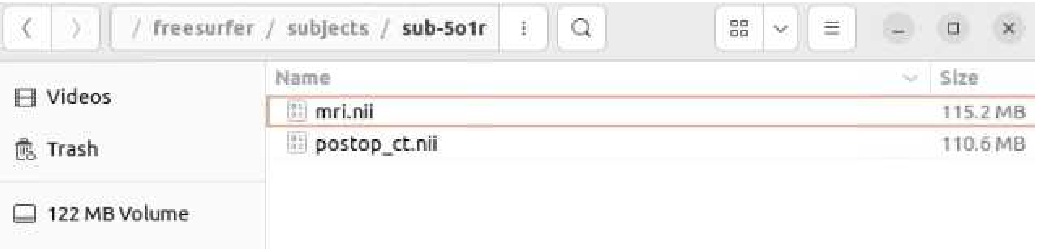

2.2 Open a terminal window and change directories within it to where you placed the mri.nii.gz file (see APPENDIX I for more info on using the terminal). Type these four commands, pressing enter after each command:

```
tcsh
setenv SUBJECTS_DIR ${PWD}
setenv SUBJECT id##_SurferOutput
recon-all -all -s $SUBJECT -i mri.nii.gz
```

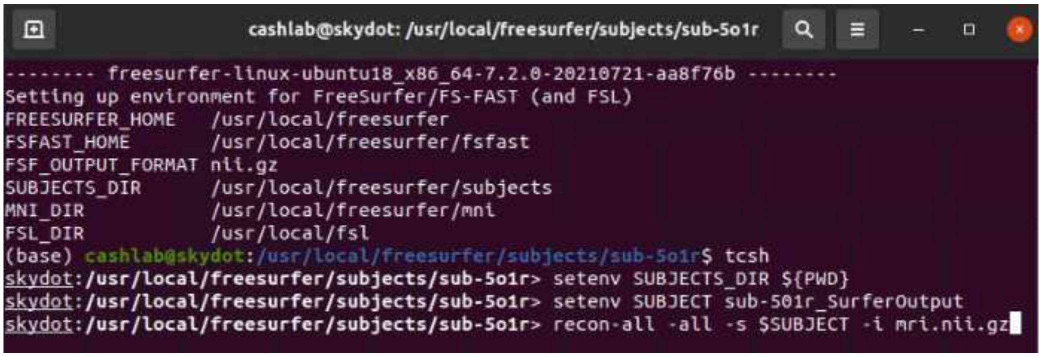

If you want to run the recon-all for a patient with grids and strips ECoG, you will need to run it with the "-localGI" flag at the end of the first command. You will also need the Image Processing Toolbox from MATLAB:

Command

```
recon-all -all -s $SUBJECT -i mri.nii.gz -localGI
```

If you want to run the recon-all with a T2, the command looks like this, where "T2.nii.gz" is the T2 or FLAIR file you wish to use to supplement your T1. You run the first three commands above the same, and replace the fourth with this:

Command

```
recon-all -all -s $SUBJECT -i mri.nii.gz -T2 T2.nii.gz -T2pial
```

2.3 Once finished, copy the pre-op NIfTI file into the *id##_SurferOutput/mri* folder and extract the file so the mri.nii(extracted) is in the *mri* folder. The extraction step can involve unzipping the file using any extraction software (e.g., WinZip, Bitser), or by right-clicking and selecting some form of “extract”.

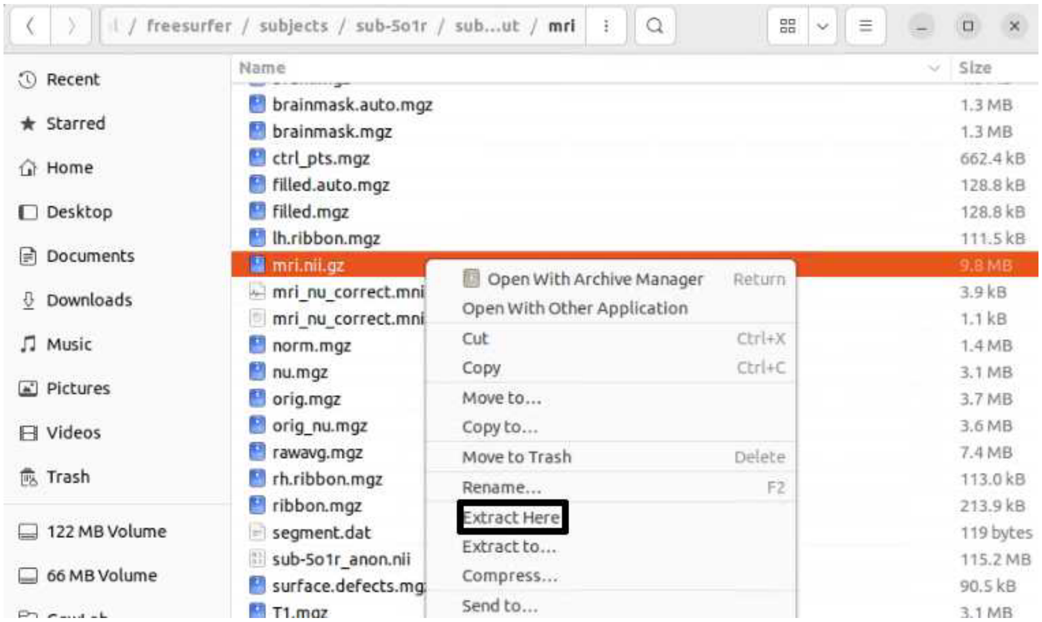

2.4 If you wish to have a better sub-thalamic segmentation, you can use a segmentation script supplied in versions of FreeSurfer 7.2 and newer. Run the first three commands from the recon-all (Step 2.2) and then run:

Command

```
segmentThalamicNuclei.sh $SUBJECT
```

This step only takes a few minutes, typically.

MRI/CT Co-Registration with Freeview

3 MRI/CT Co-Registration with Freeview

Note

This manual co-registration step can be done before or after the Surfer Output (Step 2) is made.

After installing FreeSurfer (Appendix V), this step uses a visualization tool, Freeview, to view MRIs, CTs, and many other images in 3D space. You will transform the post-op volume in this 3D viewing tool so that it aligns with the pre-op volume, and thus you can find the locations of the post-op electrodes in the 3D space of the pre-op images. When working with Freeview, you will only be manipulating the volume which is highlighted at the top left of the screen (Step 3.3, Figure). This is the most hands-on and time-intensive step and will require patience and persistence until you become skilled at it.

There are automatic co-registration approaches (iELVis: https://www.sciencedirect.com/science/article/abs/pii/S0165027017300365?via%3Dihub, LeadDBS: https://www.lead-dbs.org/, or Freesurfer tkregister: https://www.ncbi.nlm.nih.gov/pmc/articles/PMC2733527/ for instance) which could also be used.

3.1 Open a terminal, type the following command, and press enter. A window will pop up:

Command

```
freeview
```

3.2 Load the pre-op file:

- Click on File > Load Volume. A dialog box will appear. Select the pre-op NIfTI volume file in the browser. Select the “Resample to standard RAS space” checkbox to reorient to RAS coordinates. Hit “OK”.

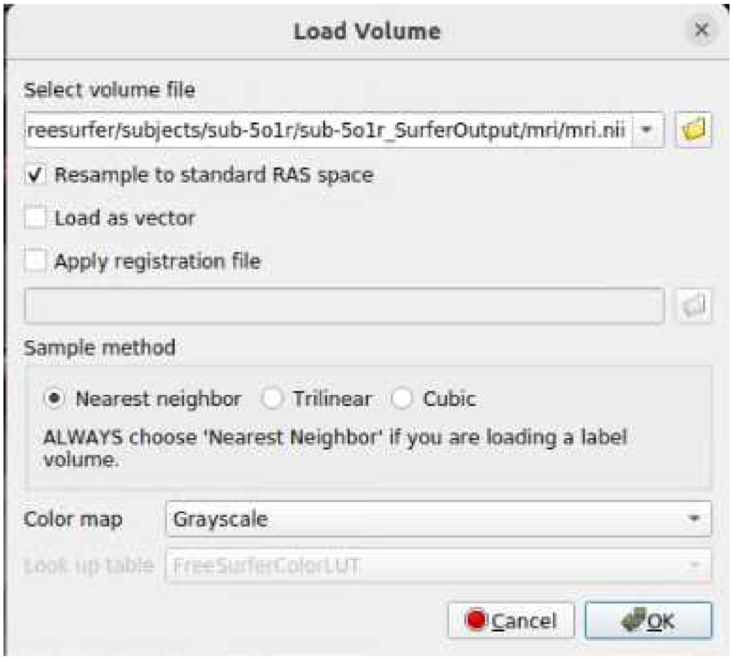

3.3 Load the post-op file:

- Click on File > Load Volume. A dialog box will appear. Select the post-op NIfTI volume file. Choose a color map (at the bottom of the box) to best visualize the electrodes and skull. We suggest the "Heat" color map. Hit “OK”.
- Further suggested adjustments:

i. Using side selection pane, adjust the min, mid, max, and offset values to best see the outline of the skull (with the post-op volume selected!).

ii. If using a post-op CT, select the “truncate” and “smooth” check boxes in this panel to remove noise in the image.

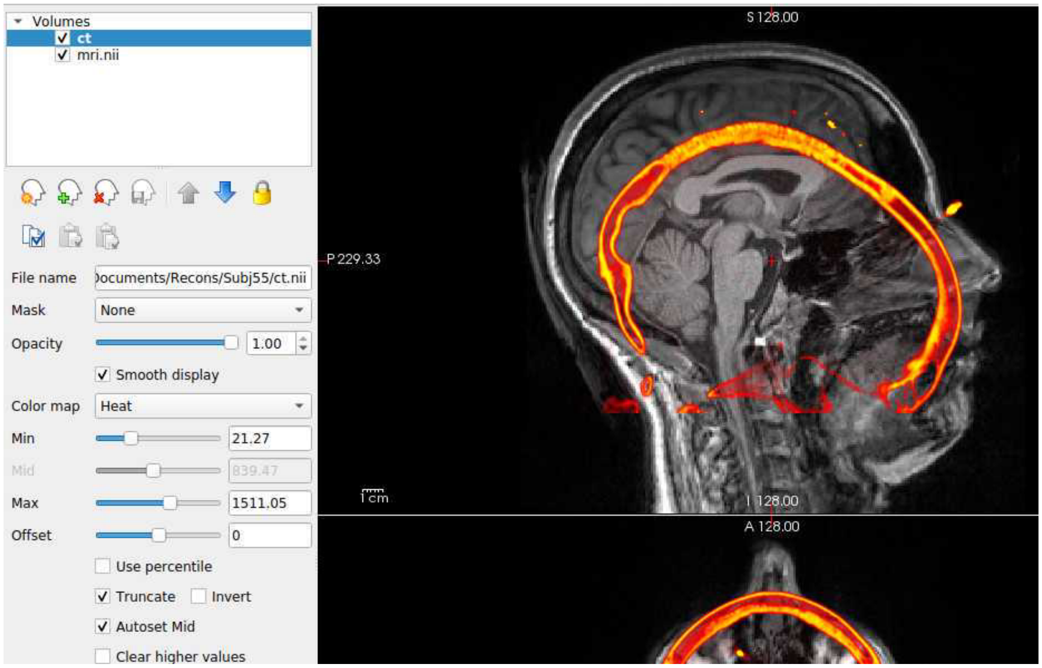

3.4 With the post-op imaging highlighted in the top left “Volumes” window (do NOT select the MRI to transform!), align the skull outline in the post-op imaging with the black of the MRI using the transform tool:

- At the top, select Tools > Transform Volume to open the tool

There are four options: Rotate, Translate, Scale, and Flip. We suggest that you practice rotating and translating the selected volume with the sliders to get a sense of what each one does and feels like to move. Scaling and Flipping are rarely ever needed.

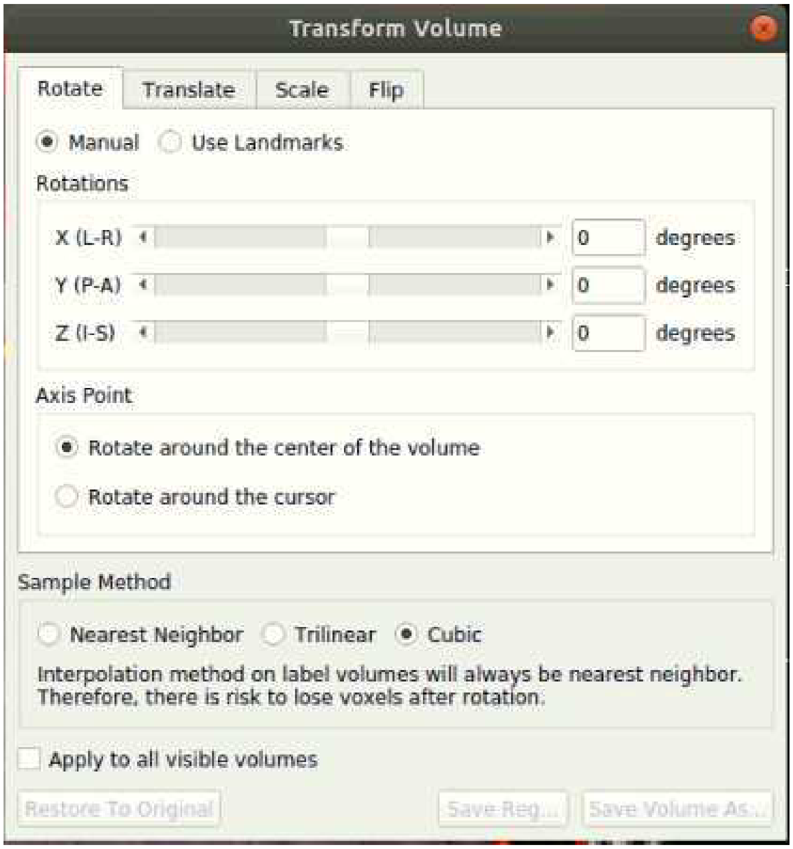

3.5 Transform the volume until the post-op image aligns with the pre-op image.

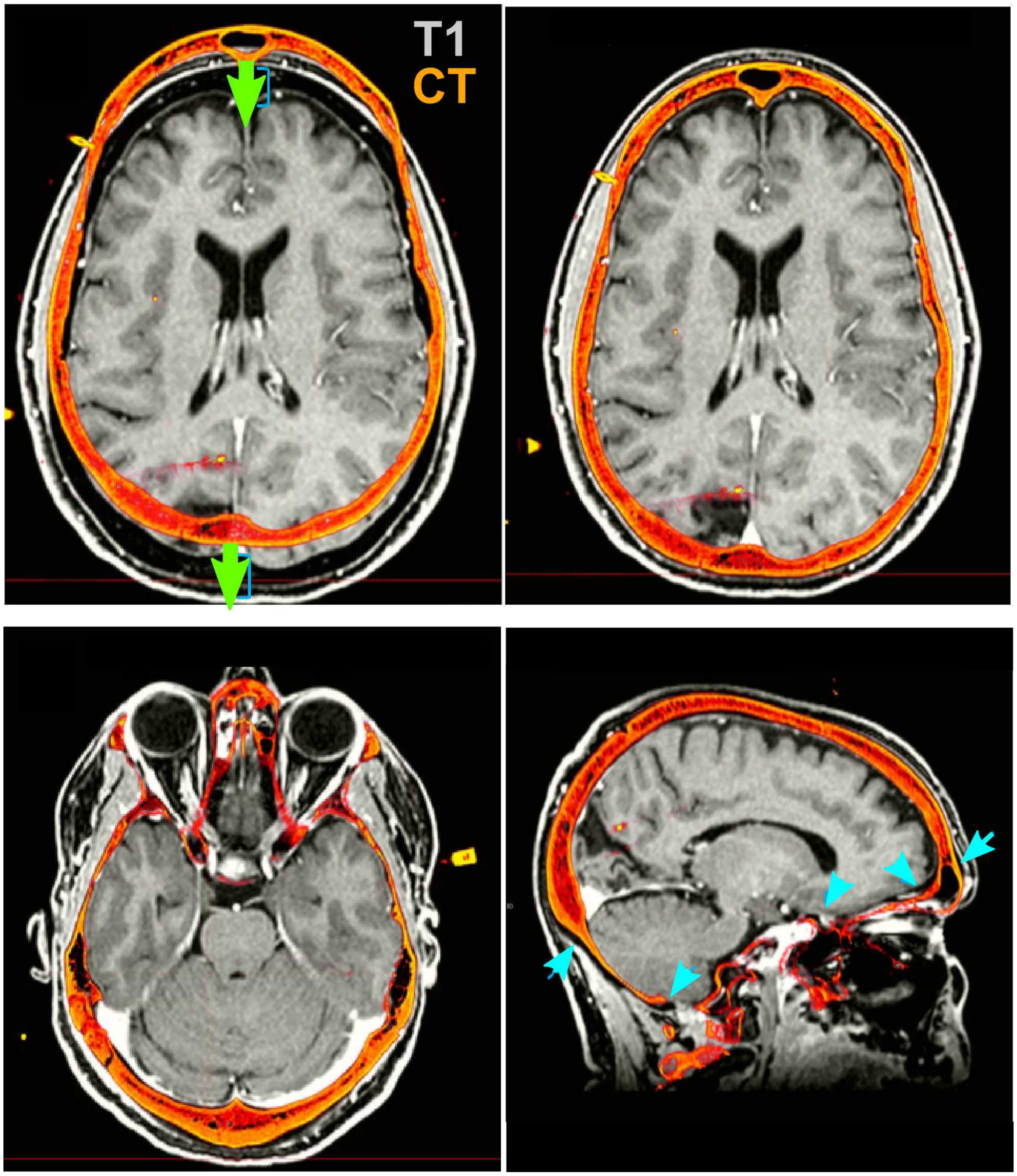
T1
CT

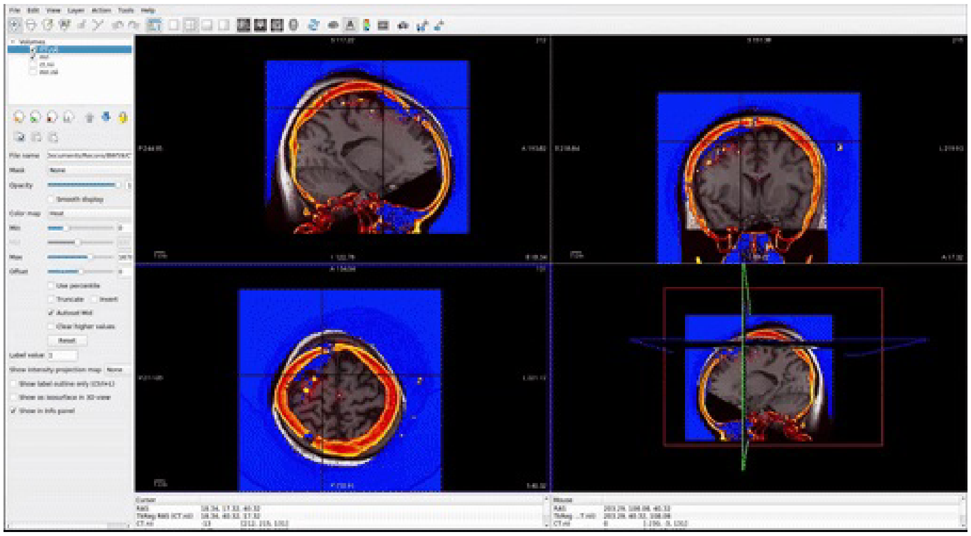

sub-03ti - Quick Coregistration Example

Note

We recommend that you write down the rotation and translation numbers that you get once aligned, in case you ever need to go back to this step in the future. We also suggest that you should only use the “scale” option in the transform tool as a last resort, since it is incredibly uncommon for this to be necessary for successful alignment.

Identifying RAS Coordinates

4 Identifying RAS Coordinates

Note

For this step, you will use Freeview to find the RAS coordinates of each electrode contact. Write down each R, A, and S coordinate in an excel spreadsheet.

4.1 Make an excel spreadsheet named ***id##***_*RAS.xlsx* with 4 columns and n+1 rows, where n is the number of electrode contacts for which you find locations in Freeview. The first column has the names of each electrode contact, and the other three are for the three axes: R, A, and S. (Right, Anterior, and Superior)

	A	B	C	D	E
1		R	A	S	
2	RTGH01	34.55	-16.41	-42.33	
3	RTGH02	37.93	-15.87	-43.42	
4	RTGH03	41.21	-15.62	-44.32	
5	RTGH04	44.5	-14.68	-45.1	
6	RTGH05	47.86	-14	-45.93	
7	RTGH06	51.37	-13.81	-46.9	
8	RTGH07	54.65	-12.79	-47.45	
9	RTGH08	58.46	-13.28	-48.33	
10	RTGH09	61.18	-12.19	-49.11	
11	RTGH10	63.17	-11.25	-49.49	
12	ROFC01	4.8	39.44	-41.89	
13	ROFC02	8	39.81	-40.52	

4.2 Highlight the pre-op mri volume layer in the top left in Freeview

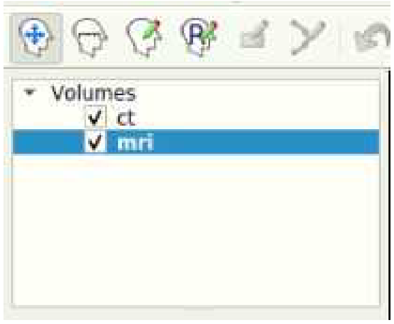

4.3 Use the crosshairs/cursor to locate each electrode. Click on the center of the contact in all three views to ensure that you have the most accurate location

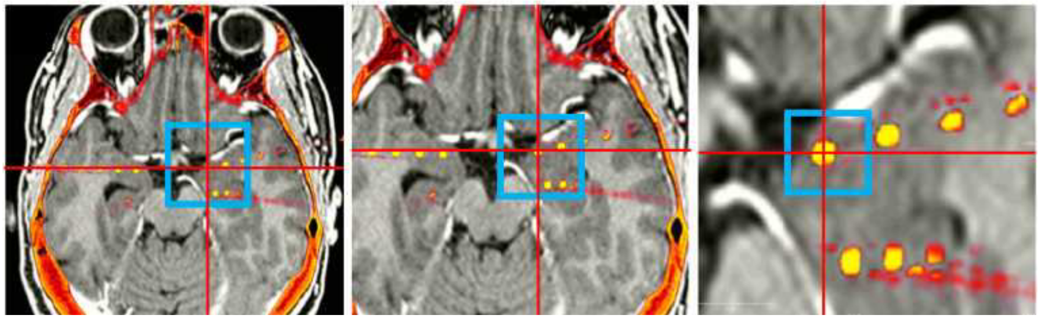

4.4 Write down the RAS from the TkReg RAS (mri) row at bottom of screen for each electrode contact. A video example of this for sub-03ti is in the Materials section.

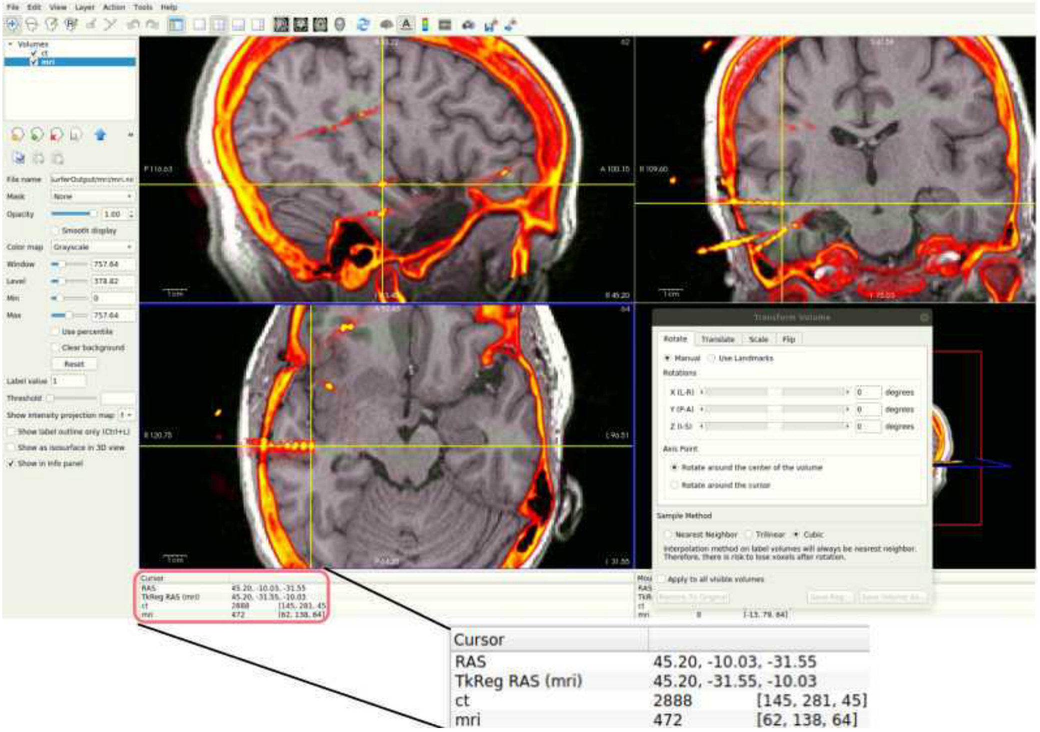

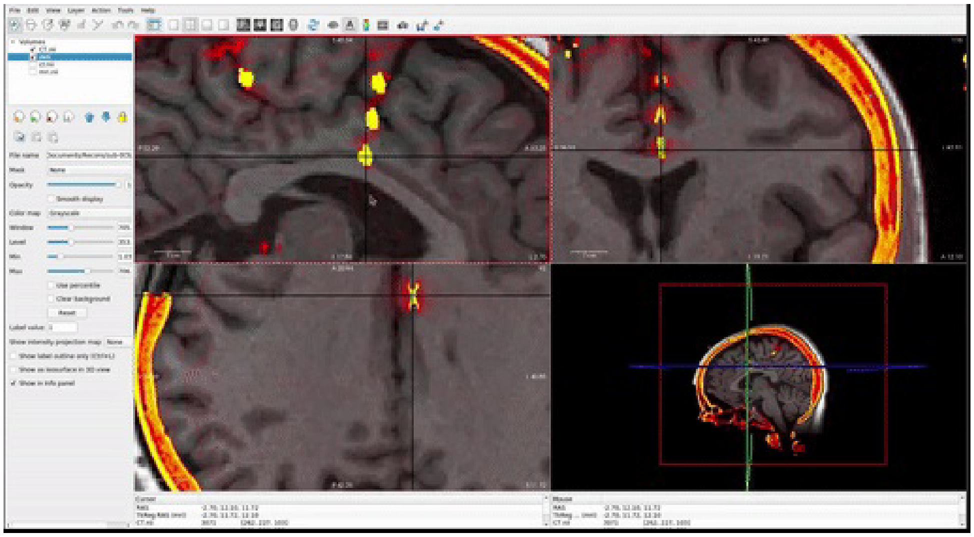

sub-03ti - Quick RAS Example

4.5 Once all RAS coordinates for all electrodes have been located, check to ensure that the orientation of the coordinates is correct using this test: look at the RAS coordinates section in

Freeview (Step 4.4 Figure, zoomed portion). The R, A, S numbers (the first, second and third numbers in the Freeview TkReg RAS row) should increase positively in the right, anterior, and superior directions, respectively. If they do not, you must account for this in coordinate transcription. There are 3 options for each of the coordinates, R, A, and S:

1. All values are in the correct orientation. Nothing additional needs to be done once you write down the value from Freeview.
2. Some of the values are switched (e.g., R may be in the S position). Test this by moving the cursor in each of the three directions and making sure the value in the correct spot changes in RAS instead of ARS or SAR. For example, moving the cursor towards the anterior of the image will result in the second value in the RAS field changing.
3. Some of the values could be inverted. This means the RAS values are in the correct order, but they may be flipped. For example, if the R value decreases when you move the cursor to the right and increases when you move the cursor to the left, then R values are inverted.

Just account for these changes in the final RAS excel sheet before moving forward. Multiply inverted columns by -1 or shift columns which were switched in the original Freeview.

Snapping the Grid to Brain Surface (only applicable for grid...

5 **Snapping the Grid to Brain Surface (only applicable for grid/strip (ECoG) electrodes. If your subject has exclusively depths (sEEG), skip to next section)**

Note

Snapping the electrode grid to the surface of the brain is necessary to account for brain movement or compression that occurs immediately after grid implantation. This brain shift can depend on the location and size of the grids used and can influence the location of the brain in the postoperative MRI or CT. Therefore, the surface of the cortex is different between the pre-operative scan used in the FreeSurfer pipeline and the post-operative scan. This difference must be accounted for by shifting the grid to the surface of the pre-op imaging, rather than relying solely on the post-op imaging.

This section requires downloading iELVis and the GitHub code (see Materials).

5.1 You should create a directory in which you place each subject's folder. We will call this the working directory (wd).

5.2 Create an **id##** folder in the wd containing:

- RAS coordinates in an Excel spreadsheet

- SurferOutput folder for the patient

For each patient, make a new copy of "***idxx**_Snap_to_Grid.m*" from the *Protocol_Snap_to_Grid.m* code file located on the GitHub page. This isn't necessary, just a recommendation.

5.3 Change the *Pt* (line 10) and the *reconPath (line 11)* variables to be the patient's name and the path to your wd folder, respectively. Also, ensure that the GitHub code and iELVis code are on the path.

```
1     %% Modular Reconstruction and Co-registration of Imaging from Implanted ECoG and SEEG Electrodes
2     %
3     % Code for Snapping Grids and Strips to Pial surface
4     %
5     % This version was completed on November 29th, 2022
6     %
7     % For questions, please post issues to the GitHub
8     %
9
10 -  Pt = 'sub-Ot3i';
11 -  reconPath = '/MyPC/Documents/Recons';
12 -  [num,txt,all]=xlsread(fullfile(reconPath, Pt, [Pt '_RAS.xlsx'] ) );
```

5.4 Next, you must create a variable which has each electrode's RAS coordinates. Please look in the *Protocol_Snap_to_Grid.m* for an example of how this is done. You can partition them up as whole electrodes or break them down into strips. For example, if you have an 8x8 grid, we usually break it down into eight 1x8 strips. Each strip will usually be handled by the algorithm better than the whole grid, though you can experiment with keeping them whole. Start the counting of the RAS_coords at 1, as in this example, and not at 2, as in the RAS file itself.

```
%% Separate each of the grids and strips into different variables
%G = RAS_coords(1:56,:);%Lateral Temporal Grid
grid1 = RAS_coords(1:8,:);
grid2 = RAS_coords(9:16,:);
grid3 = RAS_coords(17:24,:);
grid4 = RAS_coords(25:32,:);
```

Breaking up an 8x8 Grid into 1x8 Strips

	R	A	S
Grid01	7.13	26.58	26.86
Grid02	10.16	26.69	27.18
Grid03	13.22	27.05	27.9
Grid04	16.94	27.69	28.51
Grid05	19.96	28.34	29.45
Grid06	23.27	29	29.79
Grid07	26.86	29.42	30.6
Grid08	30.1	30	31.59
Grid09	33.69	30.07	32.33
Grid10	36.4	31.15	32.81
Grid11	40	31.51	33.34
Grid12	43.17	32.59	34.35
Grid13	41.91	-22.1	2.15
Grid14	43.6	-20	2.6
Grid15	45.74	-17.88	3.01
Grid16	48.3	-14.84	4.1

How the Grid Breakup looks relative to the RAS Coordinates

5.5 Ensure the *side* variable (line ~40) at the top of the next section is set to the correct hemisphere ("l" for left, and "r" for right). If you have grids and strips on both hemispheres, you will have to run this through twice, once for each side. Generally, a patient should only have grids and strips on one side, so only run this on the hemisphere on which the electrodes are placed.

5.6 Run each of the *snap2dural_energy_customized(...)* lines (lines 45+) for each electrode or grid/strip segment.

```
38     %% Code for adjusting the locations of the grids and strips
39 -   surface_path = fullfile( reconPath , Pt, [Pt '_SurferOutput'], 'surf' );
40 -   side = 'r';
41     % surftype = 'pial-outer-smoothed';
42 -   [surf.vert surf.tri]=read_surf( fullfile( surface_path, [side 'h.pial-outer-smoothed'] ) );
43
44     % We recommend running each of the following lines one at a time
45 -   [gr1_coor_snapped] = snap2dural_energy_customized(IHA,surf);
46 -   [gr2_coor_snapped] = snap2dural_energy_customized(IHP,surf);
47 -   [gr3_coor_snapped] = snap2dural_energy_customized(GS,surf);
48 -   [gr4_coor_snapped] = snap2dural_energy_customized(GI1,surf);
49 -   [gr5_coor_snapped] = snap2dural_energy_customized(GI2,surf);
```

Lines of code which snap the RAS coordinates to the surface

gr#_coor_snapped are the newly snapped coordinates.

5.7 When you run this algorithm for each electrode RAS variable you made, you will see a 3D projection of the electrodes as they snap themselves to the brain's surface. You should watch each of these carefully as they will tell you if the algorithm is functioning as desired. The

electrode contact points should be moving along a relatively straight line to the brain's surface (**FIG ii**). They shouldn't criss-cross or come out of their expected alignment (**FIG i**). If they do, decrease *MaxIter* (around line 45) in the *snap2dural_energy_customized.m* file. This can involve a lot of trial and error, but generally, if it doesn't work at default (MaxIter=50), then you should decrease it to <5. You can also check the *Tips for Customizing iELVis Code Package.docx* file for some help (see Materials).

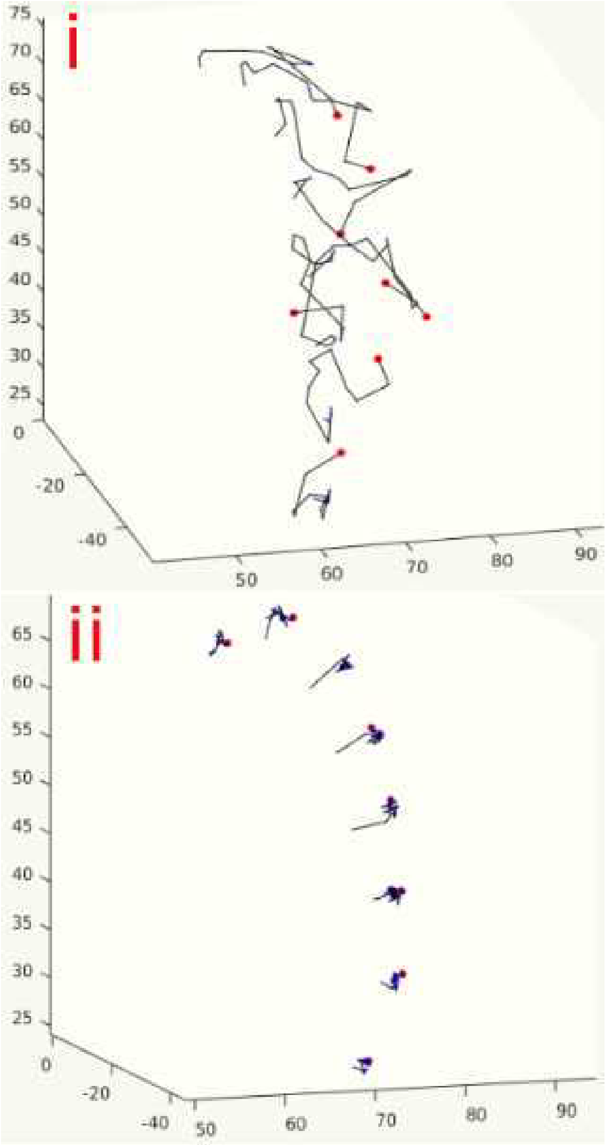

Panel **i** is a bad snap and requires changing *MaxIter.* Panel **ii** is a good snap.

5.8 Once you have the snapped coordinates, run the next two MATLAB sections which overwrites the input RAS file to have the newly snapped coordinates. This section also makes a backup so you can redo the snap if need be (*id##_RAS_bkp.xlsx*). Make sure the *RAS_coords_new*

variable (~line 55) has the electrodes in the same order that they were in the original RAS file. For example, if the input RAS has order of electrodes: GR1-64, RAH1-16, RPH1-12, STS1-16, then you should have the output *{rasElecName}_coor_snapped* in the same order in the *RAS_coords_new* variable. Plot the newly made snapped RAS and examine it carefully to ensure it is accurate. This can be done by running the first two sections of the imaging code (Step 6) with the newly snapped RAS.

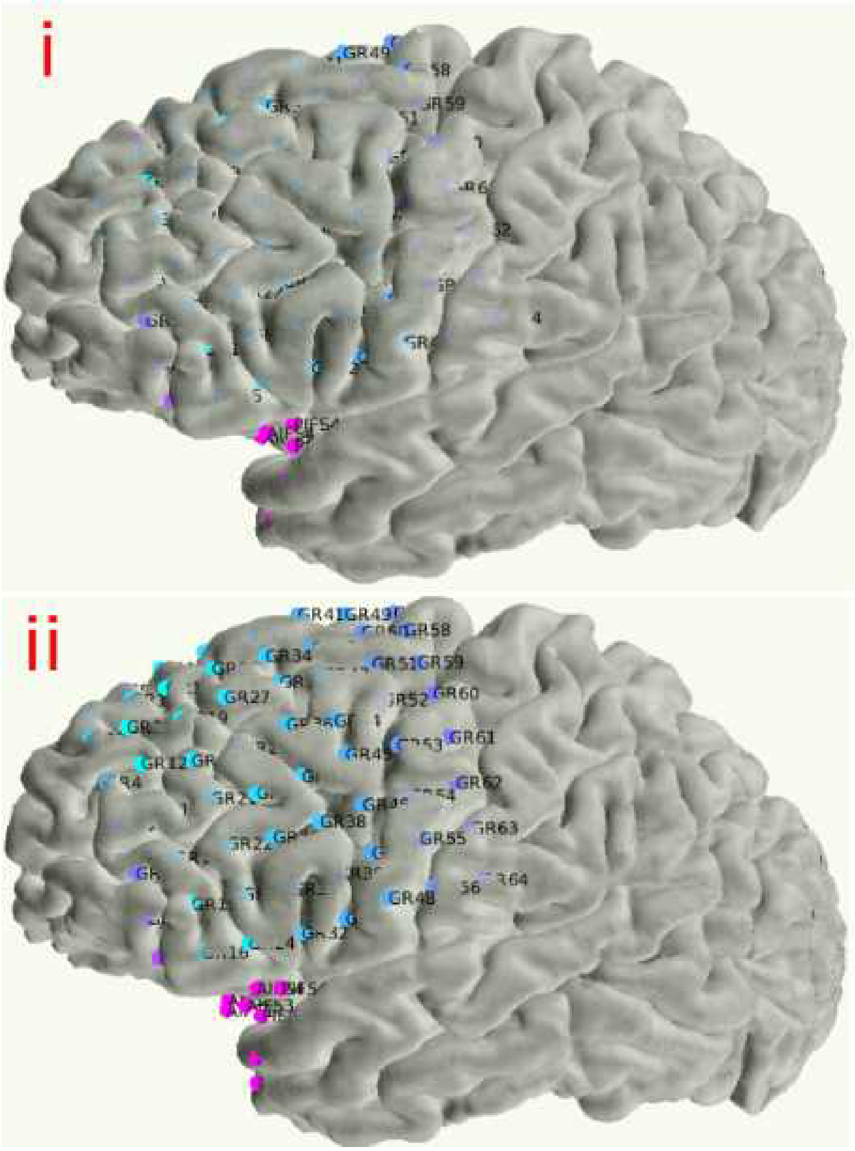

Panel **i** is an un-snapped grid, whereas Panel **ii** is snapped

Creating Images of MRI with Electrode Overlay

6 **Creating Images of MRI with Electrode Overlay**

Note

You will have to download MATLAB, Fieldtrip, and our pipeline code from GitHub in order to proceed with this step. You must initialize 3 variables before you can start. This section an output 2D and 3D views of the brain with MRI slices in order to show where the electrodes in the post-op image are in the pre-op image. You should create a directory in which you place each subject's folder. We will call this the working directory (wd).

Output examples from this section are attached in the Materials section.

6.1 Create an **id##** folder in the wd containing:

- RAS coordinates in an Excel spreadsheet
- SurferOutput folder for the patient. The mri.nii file should be in the surferoutput/mri folder.
- If you are only making 2D visuals (i.e., no SurferOutput), then you must place the mri.nii in this subject folder as well

This section will work if you have converted your files into the BIDS format (Step 8).

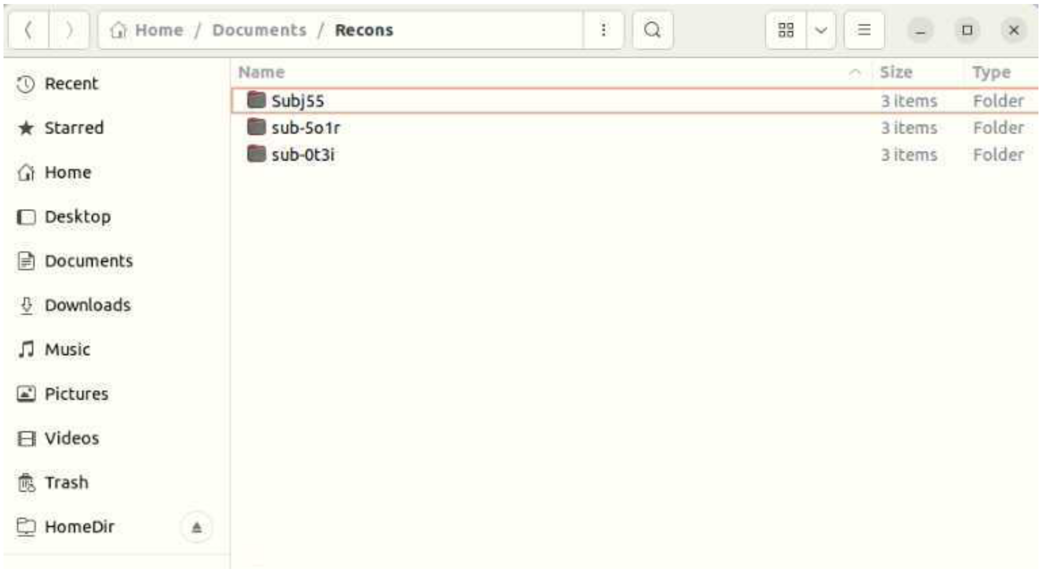

6.2 Open "Protocol_recon_code" (located in Coregistration_Figures_PreOp_PostOp) and change *wd* (line 18) to the path to your wd and *PatientName* (line 20) to your **id##.** If you have not run the FreeSurfer section (Step 2), then set *surferOutput* (line 24) to 0, and you will only get 2D visuals.

Add the paths to the Fieldtrip toolbox and GitHub pipeline code on lines 13 and 14.

```
%% Modular Reconstruction and Co-registration of Imaging from Implanted ECoG and SEEG Electrodes
% This version was completed on November 15, 2022
%
% For questions, please post issues to the GitHub
%

clear all
close

%Add the paths in this order only:
% Otherwise, functions in the fieldtrip toolbox which will make running this code
%difficult.
addpath(genpath('/home/bourbon-the-huckster/Documents/MATLAB/fieldtrip-20220202'))
addpath(genpath('/home/bourbon-the-huckster/Documents/MATLAB/Recons/Coregistration_Figures_PreOp_PostOp'))

%Path to the recons directory. This should be filled with subject folders.
%Each subject folder will have the SurferOutput and RAS Excel
wd = ['/home/bourbon-the-huckster/Documents/Recons/'];

PatientName='id##'; % name of the folder and id## in the working directory

% Setting this variable to 0 will create only the 2D visuals without
% required a SurferOutput from FreeSurfer
surferOutput = 1; % Set to 1 if you have run the Freesurfer Reconstruction
```

6.3 Run the first cell of the code, which sets variables, loads data into MATLAB, and reads the SurferOutput files you made in Step 2 to create a 3D image of the brain

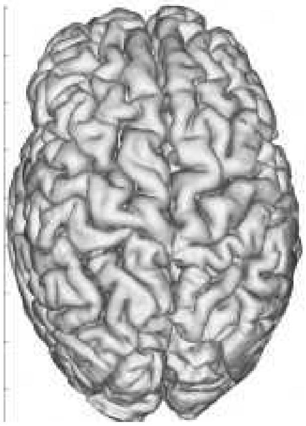

6.4 Create 3D image with electrodes by clicking "Run Section" in the second cell.

- To change opacity, change number after “facealpha” (lines 136, 138). The range is 0-1, where 1 is fully opaque. For depths, 0.3 looks the best, while 0.9-1 is best for grids/strips.
- If need be, RAS locations can be transformed on line 186-188. This is only necessary if snap does not work well.

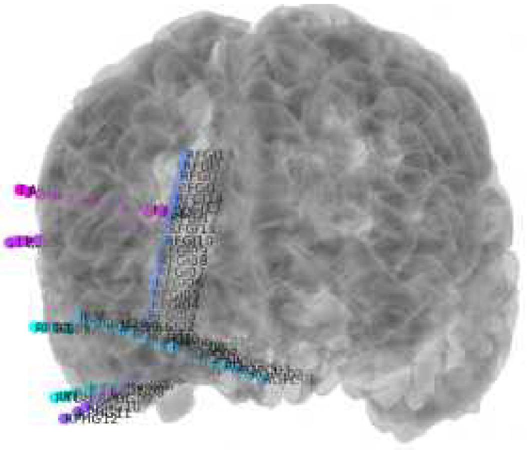

6.5 Creates Images which re-slices the MRI to capture the plane of the electrode with the coronal view along the entire depth electrode (skip for grids/strips) by clicking "Run Section" in the third section. This section involves producing coronal slices which are angled along the length of the depth electrode to ensure that every contact is shown.

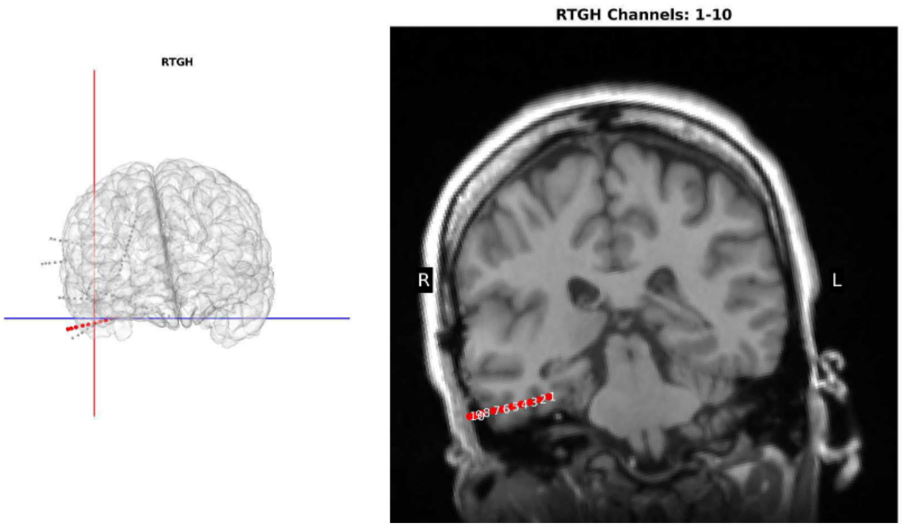

6.6 Create Images Per-Contact with MRIs and 3D Brains from all three views by clicking "Run Section" in the fourth and final section. This last section creates images for each electrode location superimposed on the MRI. This step may take many minutes depending on the number of electrodes.

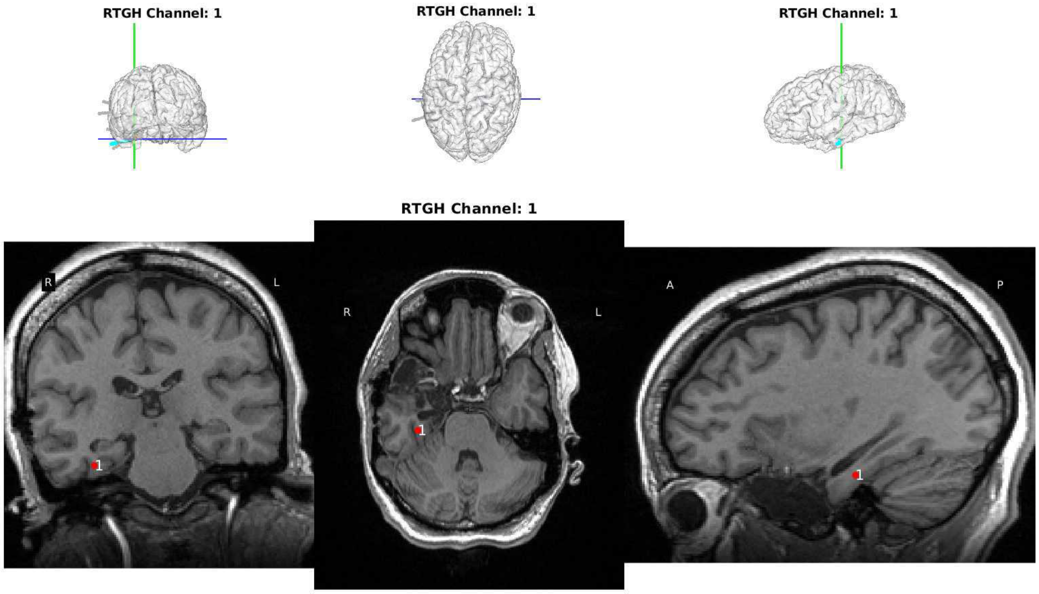

6.7 On Linux, you can open a terminal in the “Images” folder and run this command if you install ImageMagick:

Command

```
convert $(ls -v *.png) Recon.pdf
```

You can also use any PDF creation tool

Multi-Modality Visualization Tool (MMVT) and Electrode Lab...

7 Multi-Modality Visualization Tool (MMVT) and Electrode Labeling Algorithm (ELA)

Note

The Multi-Modality Visualization Tool (MMVT) is an open-source python package that converts the reconstruction output into a Blender (blender.org) file for three-dimensional visualization of electrode locations which can incorporate MRI, FreeSurfer output, RAS mapping, and even evoked potentials into the visualization. We will use the “lite” version of MMVT, which is a stripped-down version, without Blender. Please follow the guide in Appendix VII to set up MMVT-lite.

You will need a FreeSurfer Output for the patient (Step 7.3), and a RAS coordinate sheet (Step 7.4). We recommend keeping them together in a “reconstruction” folder, but it is not absolutely necessary.

Importantly, using underscores in the subject ID will causes this step to fail.

7.1

If you are trying to run this part of the protocol with grids and strips, you need to separate every column or row of the grid to make it look like a separate depth (GR_##, GRa_##, GRb_##, etc). This is similar to the grid breakup from Step 5.4.

Var1	R	A	S
GR1	0.01	68.81044279	65.6228701
GR2	-7.755509349	71	69
GR3	-15.34323393	73.9753132	65.9407503
GR4	-25.84517529	74.15453227	59.3284332
GR5	-33.37804495	73.95171674	52.6561164
GR6	-41.01314074	72.94414814	43.9785945
GR7	-44.83876651	71.5956788	35.2036288
GR8	-44.13868186	68.95937598	26.1368778
GRa9	-1	61	71
GRa10	-10	63.34358609	71.7011422
GRa11	-17.91282037	66.8690308	68.3000888
GRa12	-30	68	63.9812767
GRa13	-35.47086567	67	55.4499603
GRa14	-43.9921108	65.46558369	47.5499024
GRa15	-47.84662146	63.18885669	38.293485
GRa16	-49.54358994	61.32826058	28.1745619
GRb17	-1.399475785	53.88758783	75.0386893
GRb18	-12.28743455	56.54842497	74.2067677
GRb19	-23.46146787	57.03858673	70.8944002
GRb20	-31.54009439	57.57833643	64.1658983
GRb21	-40.68707176	56.28996301	58.3048742
GRb22	-47.56842396	55.47815997	50.4045167
GRb23	-50.96466461	53.0512569	40.2293816
GRb24	-53.06901592	52.43507442	30.3211107
GRc25	-3.33725446	44.99259761	78.347231
GRc26	-14.63089855	47.07199391	78.039921
GRc27	-25.41306303	48.22785655	75.1498534
GRc28	-35.47863874	47.79494198	66.9719149
GRc29	-44.24557788	47.75256685	59.623609
GRc30	-51	46.42148723	52
GRc31	-55.49388181	44	42
GRc32	-55	41.61996725	31.8963978

7.2 Navigate to your “mmvt_root” folder in a terminal. Activate the environment for MMVT and change directories:

Command

```
source mmvt_env/bin/activate                                  cd
mmvt_code
```

Note

You can check which shell you are using if you type "echo $0" and hit enter. If you are in the tcsh shell still, you will need to run the command as:

source mmvt_env/bin/activate.csh

From now on, make sure you are running every line of code from the “mmvt_code” folder.

7.3 First, we must parcellate the brain surfaces based on a given atlas. Run this command to start the parcellation:

Command

```
python -m src.preproc.anatomy --remote_subject_dir "Path/To/id##_SurferOutput" -s id##
```

It should take a few minutes. You’ll get a parcellation of the subject’s brain along with other surface files in “/…/mmvt_root/mmvt_blend/{**id##**}” that will let you run the next step.

Note

You can run this and the next step on multiple subjects at once, see Appendix VII for information.

The DKTatlas40 is the default atlas. To use another atlas, use the –a flag and the name of the atlas. For example, adding “-a laus125” will run the parcellation using the lausanne125 atlas. Please see Appendix VII for information about using different atlases.

7.4 Run this command to run the Electrode Localization Algorithm (ELA):

Command

```
python -m src.preproc.electrodes --remote_ras_fol "Path/To/id##_RAS Folder” --
find_hemis_manual 1 -s id## -b 0
```

-b 0 finds electrode locations for referential electrodes, -b 1 makes electrode locations for bipolar electrodes. Run as needed.

- Add --overwrite_ela 1 to overwrite files already there
- If it says: “bad subject”, it’s usually fine. This just means not 100% of the algorithm ran. It will show different error messages if something important for ELA went wrong.
- --find_hemis_manual 1 turns on the ability to find the hemispheres for each electrode manually, though this will usually only require more input if you have grids and strips. You will have to type “l” or “r” for each electrode when prompted to indicate which hemisphere of the brain the electrode is in.

The important outputs of the electrode localization step are .csv files that contain the names of the electrode contact points in the first column and the subsequent columns—save for the last two—contain the probabilities that the a parcellated area of grey matter contains electrode contact points. The files are in /mmvt_root/mmvt_blend/[**id##**]/electrodes, and they are labeled:

id##_aparc.DKTatlas40_electrodes_cigar_3_l_4.csv

Or:

id##_aparc.DKTatlas40_electrodes_cigar_3_l_4_bipolar.csv

Again, the "-a" flag will be important for specifying the atlas. Please keep the -a flag consistent between the parcellation and the ELA steps. See Appendix VII for more information.

Note

Verifying the ELA output:

- Look at probabilities for electrodes and make sure it makes sense for where they should be
- If it fails, run with ela_overwrite 1 or you must delete files in these three locations to start over:
- /home/cashlab/mmvt_root/electrodes//{**id##**}
- /home/cashlab/mmvt_root/mmvt_blend/{**id##**} + files at the bottom
- /home/cashlab/mmvt_root/electrodes_rois/electrodes

iEEG BIDS Data Formatting for Data Sharing

8 iEEG BIDS formatting of channel labels and file formats

A final step for the RAS file is to prepare the RAS channel data following FAIR practices (https://www.nature.com/articles/sdata201618) and the standard iEEG BIDS format (https://www.nature.com/articles/s41597-019-0105-7). For the iEEG BIDS formatting, manual entry to the RAS file for the electrode types (sEEG, grid, strip, or ECoG), electrode manufacturer,

per-contact surface area (exposure to the brain), electrode group (e.g., the prefix label in front of electrode number), and the brain hemisphere. These details are recommended standard practice for information per RAS file, and, in our case, we made a copy and named this file:
"(patient designation)_ElectrodeInfo_RAS.xlsx" (https://www.nature.com/articles/s41597-019-0105-7).

	R	A	S	ElectrodeTyp	Manufacture	SurfaceAreas	group	hemisphere
IHA1	-2.64	12.24	11.61	strip	AdTEch	7	IHA	L
IHA2	-2.14	13.97	22.02	strip	AdTEch	7	IHA	L
IHA3	-2.14	15.92	31.87	strip	AdTEch	7	IHA	L
IHA4	1.4	17.33	40.76	strip	AdTEch	7	IHA	L
IHA5	9.79	17.6	43.81	strip	AdTEch	7	IHA	L
IHA6	18.85	17.6	40.34	strip	AdTEch	7	IHA	L
DA1	18.27	-5.27	26.22	sEEG	AdTEch	6	DA	L
DA2	18.27	-2.73	30.92	sEEG	AdTEch	6	DA	L
DA3	18.27	-0.57	35.12	sEEG	AdTEch	6	DA	L
DA4	18.35	2.17	39.82	sEEG	AdTEch	6	DA	L
DP1	11.72	-17.87	32.31	sEEG	AdTEch	6	DP	L
DP2	13.67	-16.17	36.28	sEEG	AdTEch	6	DP	L
DP3	16.24	-14.34	41.08	sEEG	AdTEch	6	DP	L
DP4	18.55	-12.27	44.84	sEEG	AdTEch	6	DP	L
IHP1	-2.03	-21.05	33.27	strip	AdTEch	7	IHP	L
IHP2	-0.68	-26.22	41.12	strip	AdTEch	7	IHP	L
IHP3	6.26	-29.86	47.18	strip	AdTEch	7	IHP	L
IHP4	15.77	-29.52	47.18	strip	AdTEch	7	IHP	L
GS1	17.22	-46.37	37.18	grid	AdTEch	7	GS	L

Example spreadsheet of the RAS output.

The spreadsheet file along with other files can then be saved into the iEEG BIDS format, which involves following the BIDS file structure. Additional code is included (https://github.com/Center-For-Neurotechnology/Reconstruction-coreg-pipeline/tree/main/Deidentification_BIDSiEEG) that uses the bids-starter kit code (https://bids-standard.github.io/bids-starter-kit/) and tailors it for this current format.

8.1 The spreadsheet file along with other files can then be saved into the iEEG BIDS format, which involves following the BIDS file structure. Additional code is included (https://github.com/Center-For-Neurotechnology/Reconstruction-coreg-pipeline/tree/main/Deidentification_BIDSiEEG) that uses the bids-starter kit code (https://bids-standard.github.io/bids-starter-kit/) and tailors it for this current format.

For the shared example data sets, we then saved all the files into the iEEG BIDS format, placing the FreeSurfer output, the volumes per brain label (see below), and the MMVT output (see below) within the 'derivatives' folder. See the example data to follow the same data structure.

•Each participant is listed as sub-XXXX
•There are:
•per-participant files (highlighted in green)
•per-task files (highlighted in blue)
•There is the preimplant scan and postimplant scan (anat) with required .jsonfiles and both need to be defaced to deidentify the scans

•The file structure and naming matters and needs to be validated using BIDS-Validator: https://bids-standard.github.io/bids-validator/
•Information required and recommend in these files are listed here:
•https://bids-specification.readthedocs.io/en/stable/04-modality-specific-files/04-intracranial-electroencephalography.html
•And a tutorial here:
•https://bids-standard.github.io/bids-starter-kit/tutorials/ieeg.html

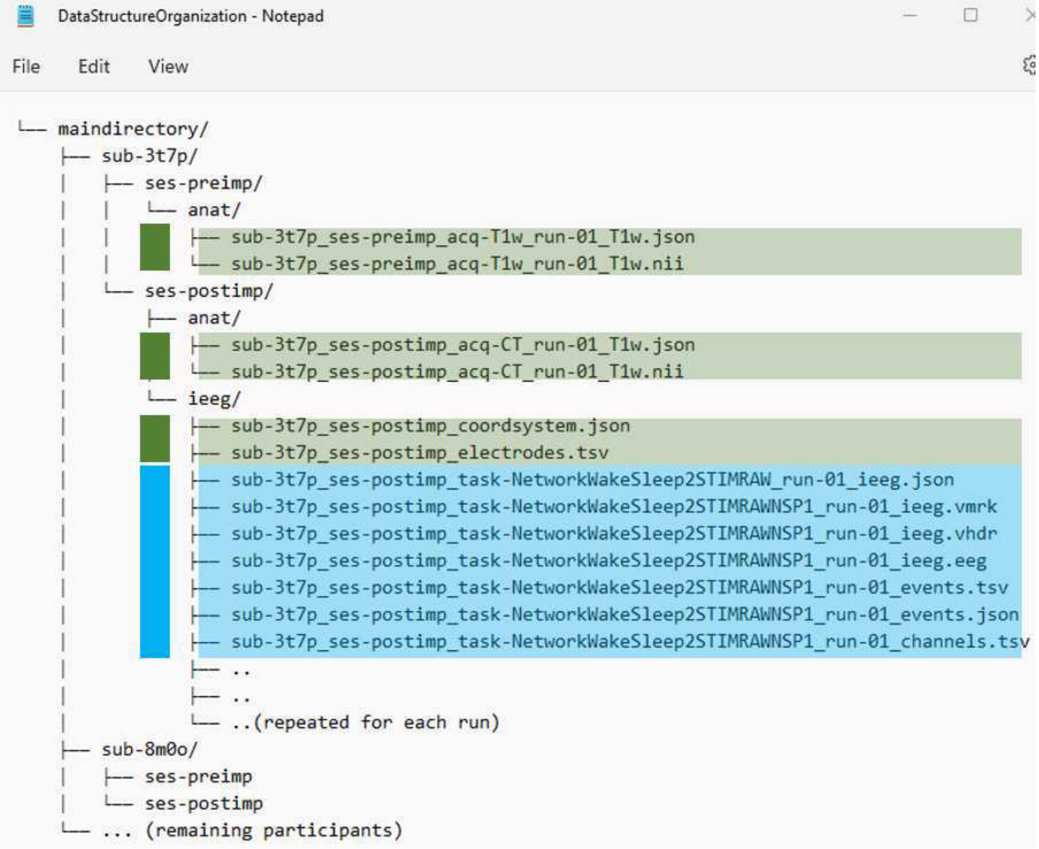

iEEG BIDS example data organization

Measurements of electrode location relative to brain featur...

9 Measurements of electrode location relative to brain features

Following electrode localization, there are a number of different measures which could be relevant to further study, including localization relative to grey and white matter, different brain regions using a volumetric approach, contact size, and contact spacing. The organization of the

FreeSurfer folders and RAS coordinates allow us to perform automatic calculations of these metrics using the current code.

A second type of labelling, electrode volume labelling (EVL), involves mapping electrode locations relative to volumes (as opposed to surfaces like ELA). To do this, we have to export the FreeSurfer parcellations to volumes, which involves using 3DSlicer (https://www.slicer.org/). The steps involve importing files from the surfer folder and then saving the volumes per brain label:

9.1 Load the brain.mgz and aparc.DKTatlas+aseg.mgzfiles (or whatever atlas you prefer) from the SurferOutput folder.

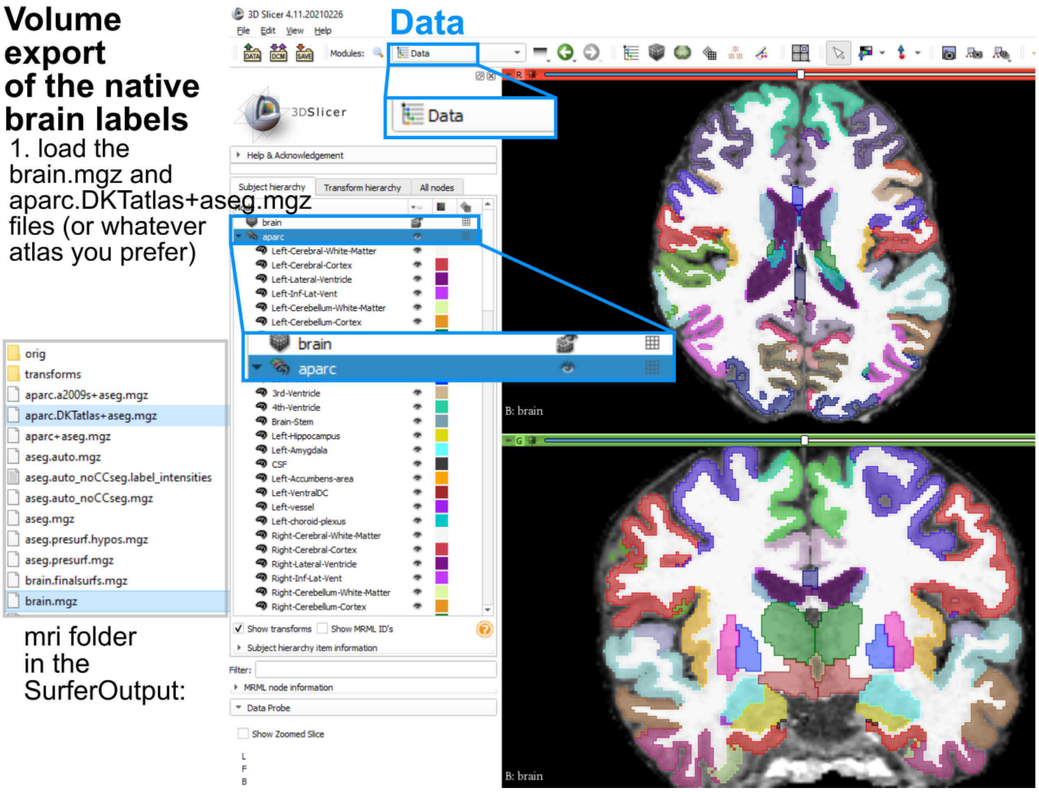

Screenshot of 3DSlicer (https://www.slicer.org/) imported aparc.DKTatlas+aseg.mgz files overlaid on the brain.mgz file from the FreeSurfer and MMVT-lite ouput (thought it can just be from FreeSurfer following parcellations (https://surfer.nmr.mgh.harvard.edu/)

9.2 View the 3D render of the parcellations.

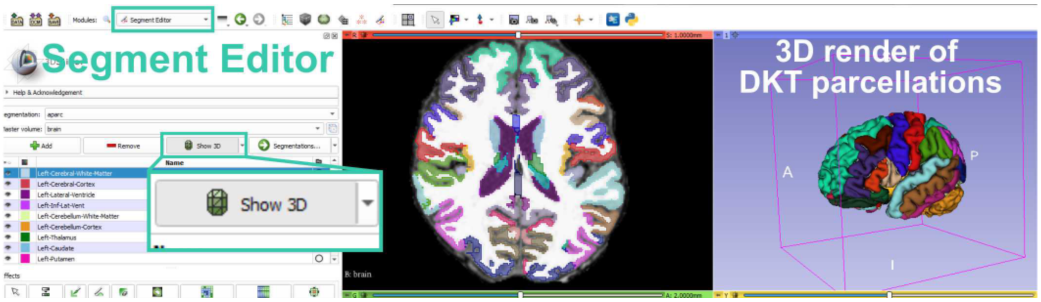

Screenshot of 3DSlicer (https://www.slicer.org/) visualizing brain region volumes as 3D structures.

9.3 Save the output volumes of the parcellations: save (export) to the selected folder.

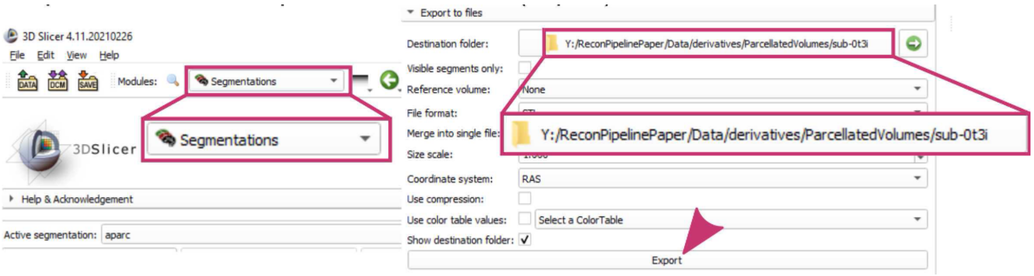

Screenshot of 3DSlicer (https://www.slicer.org/) export of volumes to .stl files

The resultant saved volumes can be used for later processing and mapping of electrode labels.

9.4 The next step involves involves running already created MATLAB code that uses the FreeSurfer, RAS, and MMVT file output structure to perform different calculations regarding electrode location, label electrodes based on the DKT40 atlas (https://mindboggle.info/data.html) or other selected brain atlas maps and produce output that matches the iEEG BIDS formatting. The code itself (Measurements_and_Labelling_RAS.m) performs the calculations and is dependent on the FreeSurfer and MMVT file structures and folder organization but, once organized, can just be run after pointing the script toward the correct participant designation. The code produces a spreadsheet (below), which includes further different measures and brain localization information. The code currently works with MATLAB 2020b onward though several of the functions can be run with older versions of MATLAB.

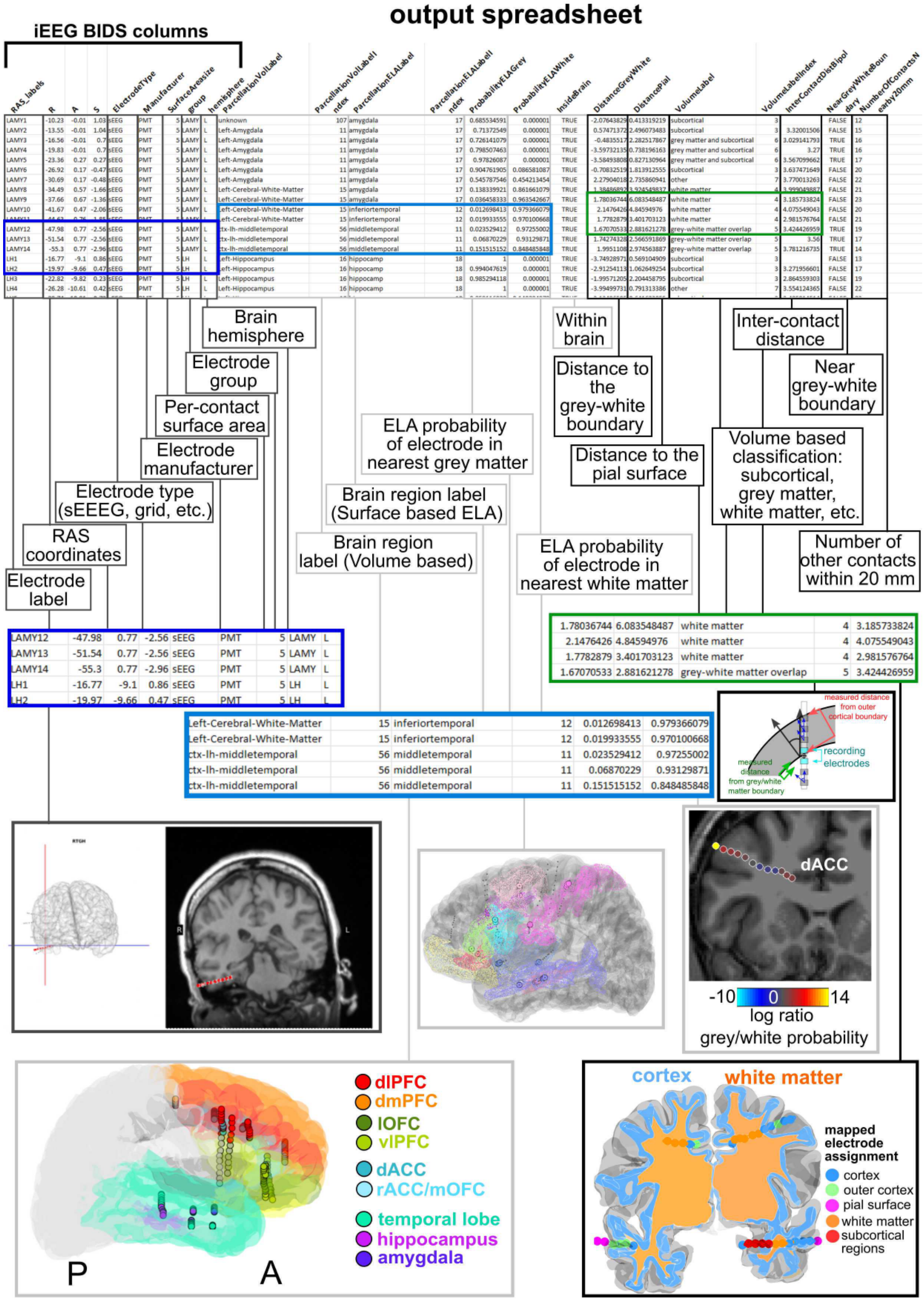

Output spreadsheet regarding measures of each contact relative to anatomical features as well as features of each contact

De-Identifying MRIs and CTs

10 To remove identifying features, in addition to de-identifying the DICOM headers or re-saving the data as NIFTI files and removing the identifying header information, with instructions and approaches to do so outlined by various other sites (https://www.fieldtriptoolbox.org/).

An additional step is to remove facial features in the MRI and CT images and save the images into a format that can be read by steps in the pipeline such as FreeSurfer. This step involves using the Fieldtrip software and the functions ft_read_mri, ft_defacevolume, and the ft_write_mri functions. The procedure involves setting the path to the downloaded Fieldtrip code (see instructions on the Fieldtrip website) followed by running the functions pointing to the files and directories where the preoperative and postoperative images were located. The ft_defacevolumefunction opens up a graphic user interface (GUI) window that allows you to change he rotation and shape of the yellow box by entering numbers into the lower right corner of the box. Once you are satisfied the box covers the facial features for the scan, close the window which will result in saved anonymized MRI which can be saved using the ft_write_mri function.

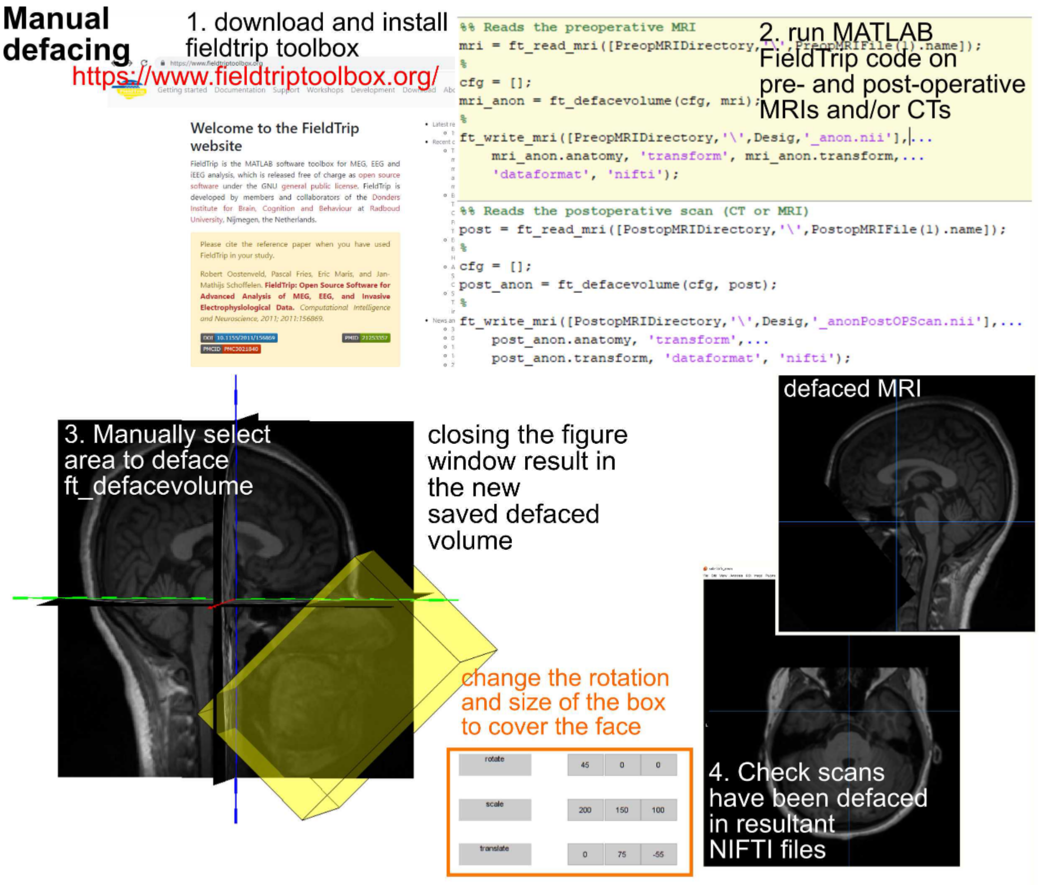

Manual defacing pipeline using Fieldtrip (https://www.fieldtriptoolbox.org/)
